# Nonlinear optoelectronic engine drives monolithic integrated photonic computing

**DOI:** 10.1038/s41377-025-01970-3

**Published:** 2025-09-04

**Authors:** Sha Zhu, Ning Hua Zhu

**Affiliations:** Institute of Intelligent Photonics, Nankai University, Tianjin, China

**Keywords:** Microwave photonics, Integrated optics

## Abstract

The rapidly growing computational demands of artificial intelligence (AI) and complex optimization tasks are increasingly straining conventional electronic architectures, driving the search for novel, energy-efficient processing paradigms. Photonic computing, which harnesses the unique properties of light to perform computation, has emerged as a compelling alternative. This perspective highlights a key advancement: a versatile nonlinear optoelectronic engine based on integrated photodetectors and micro-ring modulators (PD + MRM). This engine enables crucial functionalities like nonlinear activation and signal relay, forming a core building block for monolithic photonic processors. Its application in integrating optical Ising machines for optimization and optical recurrent neural networks (RNNs) for AI has been examined recently. The PD + MRM unit’s inherent compactness, efficiency, and on-chip reconfigurable nonlinearity address historical photonic computing challenges, signaling a shift towards more versatile and scalable monolithic photonic processors.

## Abstract (web summary)

A compact, nonlinear optoelectronic photodetectors and micro-ring modulators engine enables scalable, energy-efficient photonic processors for AI and optimization, addressing key challenges in optical Ising machines and photonic recurrent neural networks.

## Graphical abstract

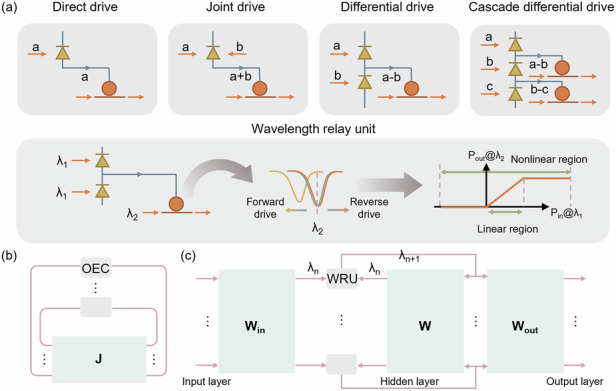

The rapid expansion of artificial intelligence (AI), machine learning, and the need to solve increasingly complex optimization problems are pushing the boundaries of traditional electronic computing. Conventional von Neumann architectures, despite decades of refinement, face mounting challenges with energy consumption and the physical limits of Moore’s Law, often termed the “power wall” and “memory wall”. This computational bottleneck is particularly acute in large-scale data centers essential for modern AI and at the network edge, where power efficiency is paramount^1^.

Photonic computing has emerged as a compelling alternative, with the potential to perform specialized tasks at significantly improved speeds and energy efficiency^2^. It achieves this by harnessing the intrinsic properties of light, such as high bandwidth, inherent parallelism, and low latency. The development of monolithic photonic processors, where complex optical systems are integrated onto a single chip, is crucial for realizing this potential, especially for dedicated tasks in optimization and AI. A critical enabler for such processors is the availability of compact, efficient, and versatile on-chip components capable of performing nonlinear functions—a historical stumbling block for photonic platforms^3^.

Recent work by Wu et al. has unlocked the full potential of the optoelectronic unit that acts as such an engine^4–6^. This unit is built upon the tight integration of a photodetector (PD) with a micro-ring modulator (MRM). This engine can be configured to perform a full suite of operations, including addition, subtraction, multiplication, and, critically, provide selectable on-chip linear and nonlinear functions. This adaptability allows the core device to be deployed in diverse computational paradigms; it is referred to as an efficient optoelectronic coupled (OEC) unit when constructing an optical Ising machine, and as a wavelength relay unit (WRU) when building an optical recurrent neural network (RNN).

This PD + MRM engine provides a rich repertoire of computational functionalities. It can perform addition and subtraction (via differential PDs), multiplication, and, crucially, nonlinear activation functions (like sigmoid or ReLU) by tuning the MRM’s operating point (Fig. 1a). The transfer function plot of the WRU clearly demonstrates its selectable linear and nonlinear regions. This localized optoelectronic interaction enables the efficient execution of these operations, offering a practical pathway to on-chip nonlinearity—a long-standing challenge for photonic neural networks. The optical-electrical-optical (O-E-O) conversion is optimized for specific nonlinear tasks, making it distinct from power-hungry analog-to-digital/digital-to-analog (AD/DA) conversions and more advantageous than all-optical nonlinear schemes that often require high optical powers or specialized materials^7,8^.

**Fig. 1:**
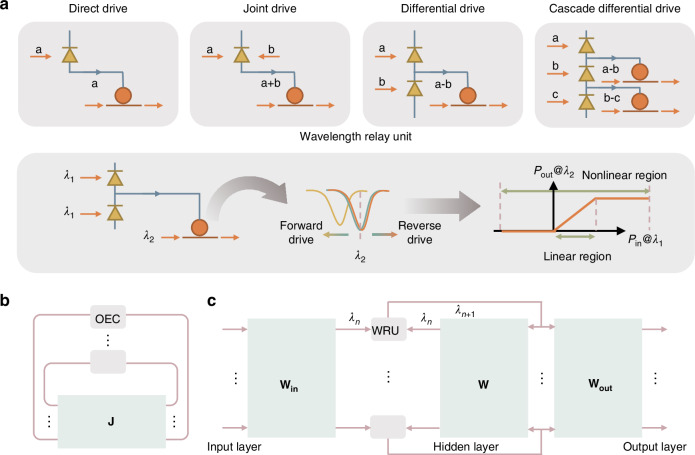
Nonlinear optoelectronic engine and its application. **a** Different driving modes of the PD + MRM engine, including direct drive, joint drive, differential drive, and cascade differential drive. The wavelength relay unit is depicted to demonstrate the operational principle of the PD + MRM engine. **b** The application of the PD + MRM engine in the optical Ising machine. **J** is the spin coupling matrix in an Ising problem. **c** The application of the PD + MRM engine in an optical recurrent neural network. **W**_**in**_ is the weight matrix for the input vector, **W** is the feedback weight matrix for the hidden vector, and **W**_**out**_ is the weight matrix for the output

The true power of this versatile PD + MRM engine lies in its ability to enable the monolithic integration of diverse and computationally intensive processors. A prime example is the optical Ising machine, a specialized computer designed to solve complex combinatorial optimization problems^6^. Wu et al. demonstrated a monolithically integrated four-spin Ising machine using these OEC (PD + MRM) units as optoelectronic coupled oscillators, which provide the essential nonlinear dynamics for the system to settle into its ground state (Fig. 1b). The system integrates a custom-designed Mach-Zehnder Interferometer symmetric coupling matrix with these OEC units. This four-spin machine achieved a rapid spin evolution time of 150 ns and a 1.71 ns round-trip time, successfully solving various four-spin problems. This monolithic approach significantly reduces latency and energy overheads compared to previous hybrid Ising machines that relied on off-chip electronic processing.

Concurrently, the same fundamental PD + MRM building block, configured as a WRU, has enabled the monolithic integration of optical Recurrent Neural Networks (RNNs), which are vital for AI tasks involving sequential data. Wu et al. introduced an asynchronous computing paradigm for an on-chip optical recurrent accelerator based on wavelength encoding and these WRUs (Fig. 1c)^5^. The WRU’s operational principle involves an “information relay based on O-E-O conversion”. An asynchronous input wavelength is detected by photodetectors, which then drive the MRM supplied by a light source with a separate wavelength. Each WRU, with its internal O-E-O conversion and selectable transfer functions, acts as a neuron. This optical RNN integrates hundreds of linear and nonlinear computing units into a compact 10 mm² footprint, showcasing superior energy efficiency and low latency.

Despite this significant progress, the journey towards practical application is paved with challenges. Scaling these systems from a few spins or tens of neurons to the thousands needed for real-world problems is a primary hurdle. Furthermore, the intrinsic bandwidth of the PD + MRM structure itself presents a critical bottleneck. The photodetector generates a photocurrent, leading to a trade-off in driving the modulator. Operating the modulator using carrier injection allows for efficient PD driving, but the speed is inherently limited (for example, to around 100 MHz)^9,10^. Conversely, carrier depletion is necessary for higher speeds, but this requires a large parallel resistor to convert the photocurrent into a sufficient driving voltage^11^. This large resistance, however, significantly increases the system’s RC time constant, which can severely limit the overall bandwidth and potentially negate the speed advantages over the carrier injection approach. Addressing these device-level bandwidth limitations, alongside system-level challenges will be crucial for developing practical higher-level integration strategies.

In conclusion, the development of the PD + MRM-based optoelectronic engine represents a significant step forward in photonic computing. Its ability to provide versatile nonlinearity, compactness, and efficiency on-chip addresses long-standing hurdles and opens new avenues for creating a new generation of monolithic processors for optimization and AI.
